# Membrane-associated gamma-glutamyl transferase and alkaline phosphatase in the context of concanavalin A- and wheat germ agglutinin-reactive glycans mark seminal prostasome populations from normozoospermic and oligozoospermic men

**DOI:** 10.1080/03009734.2019.1690603

**Published:** 2019-11-27

**Authors:** Tamara Janković, Sanja Goč, Ninoslav Mitić, Jelena Danilović Luković, Miroslava Janković

**Affiliations:** Institute for the Application of Nuclear Energy, University of Belgrade, INEP, Zemun, Serbia

**Keywords:** Alkaline phosphatase, gamma-glutamyl transferase, lectin-affinity chromatography, seminal prostasomes, surface glycans

## Abstract

**Background:** Human seminal prostasomes are intrinsically heterogeneous extracellular vesicles (EVs) whose composition is, additionally, influenced by different physiological conditions. Aiming at the molecular properties of the prostasomal surface exemplified by glycan compositions as a possible distinction factor, we applied lectin-affinity chromatography (LAC) as a new tool for their separation. Since glycans, generally, exhibit various biological activities, introduction of glyco-parameters as reference could upgrade standardization of EVs isolated by different methods and intended for use in biomedicine.

**Methods:** Preparations of seminal prostasomes from normozoospermic (sPro-N) and oligozoospermic (sPro-O) men were subjected to LAC on concanavalin A (Con A) and wheat germ agglutinin (WGA) columns. Prostasomes recovered in LAC-separated fractions were characterized according to the distribution of selected markers: gamma-glutamyl transferase (GGT), alkaline phosphatase (ALP), tetraspanin CD63, and total protein/glycoprotein composition.

**Results:** Two CD63-immunoreactive populations exhibiting prostasome signature bands but differing in GGT activity and surface glycans were separated on the WGA column. Additional populations having distinct profiles of total glycoproteins and which can be tracked down by ALP activity were enriched on the Con A column. WGA-separated populations were similar in sPro-N and sPro-O, whereas Con A-separated ones were strikingly different.

**Conclusions:** Membrane-associated gamma-glutamyl transferase and alkaline phosphatase in the context of Con A- and WGA-reactive glycans mark seminal prostasomes populations from normozoospermic and oligozoospermic men.

## Introduction

Prostasomes, extracellular vesicles (EVs) originating from the prostate, exhibit distinct heterogeneity regarding molecular composition, size, and internal morphology. It is thought that at least one population of prostasomes corresponds to true exosomes, i.e. they originate from late endosome pathways (1–4). In this study we aimed to explore molecular properties of the prostasomal surface exemplified by its glycan composition leading to progress in separation and purification of various populations. To achieve this goal, a lectin-affinity chromatography (LAC) format was evaluated as a tool using seminal prostasomes from normozoospermic and oligozoospermic men. Nowadays, immunoaffinity-based approaches exploiting available antibodies to different markers/cell-specific proteins have been used for the identification/purification of EVs (5–8). In contrast to antibodies, lectins—as glycan-binding proteins—are not given the attention they deserve and consequently have rarely been used for specific purposes (9,10). Thus, by applying selected lectin-affinity matrices, we aimed at a more complete distinction of existing glycan patterns, generally present/shared on putative prostasomal populations mixed in seminal plasma.

Glycans are distinct components of EVs, being distributed at their membrane surface as an integral part or associated in different modes but also in their cargo (10,11). Available experimental data suggest that, in addition to the common glycan signature of EVs, there are specificities related not only to biogenesis but also to cell physiology (12–14). In this way, cell-originated glycoproteins and those derived from the extracellular environment constitute unique molecular landscapes (12,15–18). These are still not properly recognized in research on EVs from both basic and applied aspects (19).

Surface-associated mannosylated glycans were recently reported to contribute to differences between prostasomes from normozoospermic and oligozoospermic men (20). In this study, in addition to the lectin concanavalin A (Con A) (which binds mannosylated structures), wheat germ agglutinin (WGA) (a sialic acid/GlcNAc-binding lectin) was chosen as the capturing ligand in LAC. Similarly to Con A, WGA was also shown to bind to seminal prostasomes from normozoospermic men but to react differently with non-EV material (20,21). All this makes it suitable to complement the capacity of Con A to extract prostasomal populations presumed to differ in surface glycosylation. Thus, LAC-separated prostasomes were characterized according to co-distribution of standard markers for EVs with selected glycan patterns, including activity of the glycoprotein enzymes: gamma-glutamyl transferase (GGT) and alkaline phosphatase (ALP). They are known to be associated with the prostasomal membrane, but have not been used for this purpose so far (22–24). Having in mind the intrinsic glycan properties which make EVs a heterogeneous analyte, introducing surface glyco-parameters is meaningful in terms of traceability of a particular procedure and suitable as a reference for comparison of isolates obtained by different methods.

## Material and methods

### Material

Monoclonal anti-CD63 antibody (clone TS63) was from Abcam (Cambridge, UK); 3,3′,5,5′-tetramethylbenzidine (TMB), bovine serum albumin (BSA), methyl-alpha D-mannopyranoside, and N-acetyl-D-glucosamine were from Sigma (St Louis, MO, USA). Biotinylated goat anti-mouse IgG, biotinylated plant lectins: Con A, WGA, the Elite Vectastain ABC kit, and Agarose-bound WGA were from Vector Laboratories (Burlinghame, CA, USA). Sephadex G 200, CNBr-activated Sepharose 4B, and Con A were from Pharmacia AB (Uppsala, Sweden). The silver stain kit and SDS-PAGE molecular mass standards (broad range) were from Bio-Rad (Hercules, CA, USA). Nitrocellulose membrane and Pierce ECL Western Blotting Substrate were from Thermo Scientific (Rockford, IL, USA). Microwell plates were from Thermo Scientific (Roskilde, Denmark).

### Human semen samples

This study was performed on leftover, anonymized specimens of human semen taken for routine analysis. Since existing human specimens were used, the investigation is not considered research on human subjects and was approved by the institutional ethics committee according to guidelines (No. 02–832/1) that conform with the 1975 Helsinki Declaration (revised 2008). Sperm parameters were assessed according to the recommended criteria of the World Health Organization (released in 2010), concerning numbers, morphology, and motility. Sperm cells and other debris were removed from the ejaculate by centrifugation at 1000 × *g* for 20 min.

### Isolation of prostasomes from human seminal plasma

Prostasomes were isolated from two pools of seminal plasma (SP) from normozoospermic men and two pools from oligozoospermic men. Each pool consisted of 10 individual samples. The samples (10 ml each) were pooled to average out the heterogeneity of individual samples. Prostasomes were isolated from SP according to the protocol of Carlsson et al. (1) modified by using CD63-immunoreactivity to indicate the presence of EVs. Briefly, SP pools were differentially centrifuged at 10,000 × *g* (30 min) and 100,000 × *g* (1 h) on an Optima L-90K ultracentrifuge (Beckman Coulter, Indianapolis, IN, USA). The final pellet, enriched in prostasomes, was additionally washed at 100,000 × *g* (1 h) and resuspended in 1 ml 0.05 M Tris-HCl buffer, pH 7.6. It was further purified by gel filtration on Sephadex G 200, and elution of prostasomes was tracked down by CD63, an EVs marker. Immunoreactive fractions were pooled, centrifuged at 100,000 × *g* (1 h), and isolated prostasome pellets were used for further analysis.

### Separation of seminal prostasomes by lectin-affinity chromatography (LAC)

Seminal prostasomes from normozoospermic men (sPro-N) and oligozoospermic men (sPro-O) were separated by affinity chromatography on columns with immobilized plant lectins: Con A and WGA. Column bed volume was 5 ml for Con A and 2 ml for WGA, and loading sample volumes were 0.3 ml. After incubation for 2 h at room temperature, non-bound fractions were eluted from the Con A column with 0.05 mol/L acetate buffer (pH 6.0) supplemented with 0.05 mol/L NaCl, 1 mmol/L CaCl_2_, MgCl_2_, and MnSO_4_, while the WGA column was eluted with 0.05 mol/L PBS, pH 7.2. The bound fractions were specifically eluted after addition of competitive sugars: 0.2 mol/L methyl-alpha D-mannopyranoside (for Con A) and 0.2 mol/L N-acetyl-D-glucosamine (for WGA). Finally, the tightly bound fractions were eluted with 0.1 mol/L borate buffer, pH 8.4, for the Con A column and 0.2 mol/L acetic acid for the WGA column. Each lectin-separated fraction (non-bound, sugar-eluted, and tightly bound) was concentrated by centrifugation at 100,000 × *g* (2 h), to recover EVs. They were further characterized using electron microscopy, immunodot blot, solid-phase lectin-binding assay, and electrophoresis. Estimation of gamma-glutamyl transferase (GGT) and alkaline phosphatase (ALP) activity was performed using GGT and ALP colorimetric assay kits (Bioanalytica, Madrid, Spain), according to the manufacturer’s instructions on Biosystems A25 (Barcelona, Spain). One unit of GGT activity is defined as the amount of enzyme that produces 1 μmol of p-nitroanilide per min, from L-γ-glutamyl-p-nitroanilide in the presence of glycyl-glycine, at pH 8.25. The rate of release of p-nitrophenol from 4-nitrophenol phosphate in 2-amino-2-methyl-1-propanol, at pH 10.4, is a measure of alkaline phosphatase activity and expressed as units per liter (U/L).

### Immunodot blot

Immunodot blot of EVs was performed as previously established (25). A nitrocellulose membrane was dotted with 2 µL of corresponding LAC-separated fractions and incubated with the following: (i) blocking solution containing 1% BSA for 2 h at room temperature; (ii) mouse monoclonal antibody to CD63 (5 μg/mL) diluted in 0.5% BSA/PBS for 18 h at 4 °C; (iii) biotinylated goat anti-mouse IgG (0.75 μg/mL) for 1 h at room temperature; and (iv) avidin/biotinylated-horseradish peroxidase (HRP) for 30 min at room temperature. Between each step, the nitrocellulose membrane was rinsed five times with 0.05 M PBS, pH 7.2, for 5 min. Blots were visualized using Pierce ECL western blotting substrate. Control (omitting the primary antibody) gave no visible reactions.

### Solid-phase lectin-binding assay

Concentrated LAC-separated fractions were immobilized on microwell plates in 0.05 M carbonate buffer pH 9.5 (50 µL/well) and incubated for 18 h at 4 °C. The lectin-binding assay was then performed as previously described (26). Briefly, biotinylated plant lectins (50 µL/well; 0.5 µg/mL) were added and allowed to react for 1 h at room temperature. After washing with 0.05 M PBS, pH 7.2 (3 × 200 µL), 50 µL/well of avidin/biotin-HRP complex (prepared according to the manufacturer’s instructions) were added followed by incubation for 30 min, at room temperature. After incubation, the plates were rinsed and developed using 50 µL/well TMB/H_2_O_2_ solution. The reaction was stopped after 10 min with 50 µL/well 0.16 M sulphuric acid. Optical density was read at 450 nm using a Wallac 1420 Multilabel counter Victor3V (Perkin Elmer, Waltham, MA, USA).

### SDS-PAGE

Proteins were resolved on 10% separating gel with 4% stacking gel under denaturing and reducing conditions (27) and stained with silver nitrate, using a silver stain kit (Bio-Rad) according to the manufacturer’s instructions. The gel was calibrated with SDS-PAGE molecular weight standards (broad range).

### Transmission electron microscopy (TEM)

TEM was performed as described previously (26). Samples were applied to the Formvar coated, 200 mesh, Cu grids by grid flotation on 10 μL sample droplets for 45 min at room temperature. This was followed by fixation (2% paraformaldehyde, 10 min); washing (PBS, 3 × 2 min); post-fixing (2% glutaraldehyde, 5 min), and a final wash with distilled water. Grids were then air-dried, and images were collected using a Philips CM12 electron microscope (Philips, Eindhoven, The Netherlands).

## Results

### Lectin-affinity chromatography of seminal prostasomes from normozoospermic men

**Figure 1.**
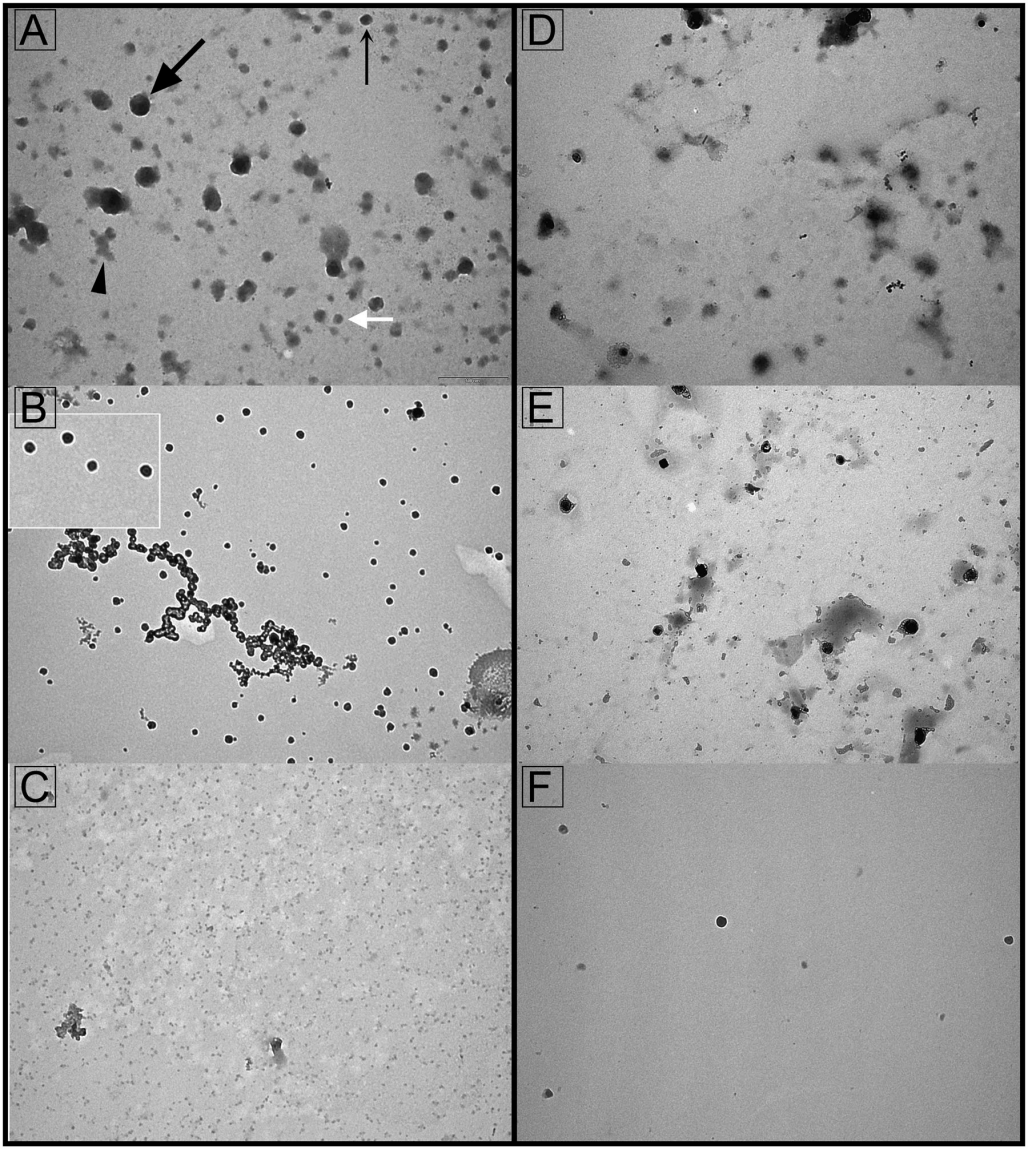
Transmission electron microscopy of seminal prostasomes from normozoospermic men separated by lectin-affinity chromatography. Micrographs of vesicles (70–170 nm) in a concanavalin A (Con A)-non-bound fraction (A). A dense vesicle (thin arrow), a dark vesicle (thick arrow), a light vesicle (white arrow), and amorphous material (arrowhead) can be clearly observed. Single (50–90 nm) or aggregated vesicles were present in a Con A-bound fraction (B). The insert shows the typical cup-shape appearance enlarged. Micrographs of vesicles (50–100 nm) in wheat germ agglutinin (WGA)-non-bound fraction (D) and vesicles (50–100 nm) in a WGA-bound fraction (E). Vesicles in the high-pH-eluted fraction from Con A column (C) and low-pH-eluted fraction from WGA column (F) were scarce.

The presence of EVs in the LAC-separated fractions was initially inspected by TEM (Figure 1). Dense vesicles having a characteristic cup-shaped appearance as well as dark round vesicles were present in non-bound (Figure 1(A,D)) and/or sugar-eluted fractions (Figure 1(B,E)) irrespective of the columns used. Light vesicles were rare. Amorphous material was more pronounced in the non-bound fractions than in the sugar-eluted fractions. The high pH-eluted fractions from Con A column (Figure 1(C)) and low pH-eluted fractions from WGA column (Figure 1(F)) were not considered further due to the low abundance of vesicles.

**Figure 2.**
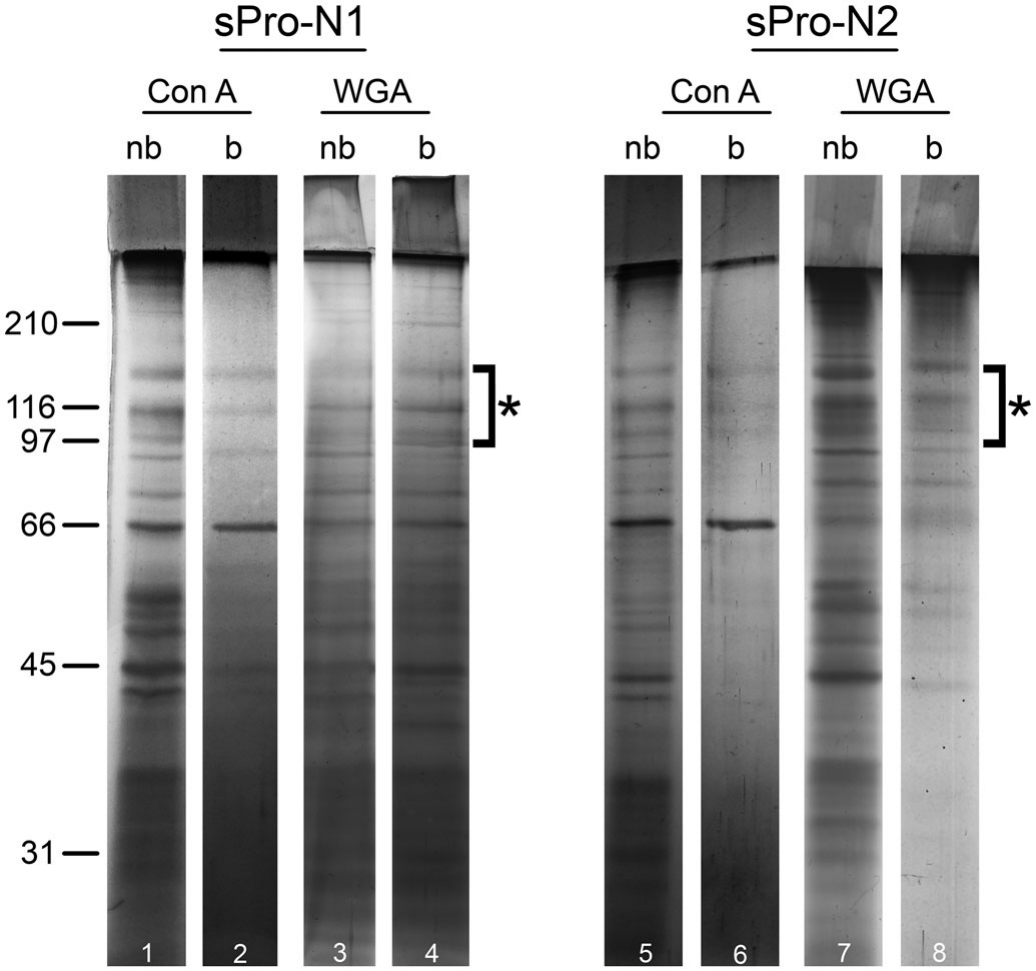
Protein composition of seminal prostasomes from normozoospermic men separated by lectin-affinity chromatography. Seminal prostasomes separated by lectin-affinity chromatography were resolved on 10% SDS-PAGE under reducing and denaturing conditions and stained with silver. Representative total protein patterns of non-bound (nb) and bound (b) fractions separated on a Con A column and on a WGA column were shown. Samples were loaded as isolated (equal volume/line to reflect yield), and total protein amounts were: line 1 (32 μg), line 2 (9 μg), line 3 (17 μg), line 4 (11 μg), line 5 (18 μg), line 6 (5 μg), line 7 (14 μg), line 8 (12 μg). Characteristic prostasome-associated bands in the region of 90–150 kDa are marked (asterisk). Numbers indicate the position of molecular mass standards (kDa). sPro-N1 and sPro-N2: different isolates of seminal prostasomes from normozoospermic men.

The electrophoretic pattern of non-bound fractions separated on Con A (Figure 2, lines 1–5) and WGA columns (Figure 2, lines 3–7) as well as the WGA-bound fraction (Figure 2, lines 4–8) revealed three prostasome-specific bands at 150–90 kDa. In the Con A-bound sugar-eluted fraction (Figure 2, lines 2–6), prostasome signature bands were at the limit of detection. Bands below 66 kDa were present in all fractions, but their electrophoretic patterns were different.

Analysis of the prostasome surface-associated enzymes, GGT and ALP, and tetraspanin CD63 revealed specific patterns of distribution as common/distinct for a particular Con A- and WGA-separated fraction (Table 1). Thus, the specific activity of GGT in Con A-separated fractions was higher in the non-bound fraction than in the sugar-eluted one. In contrast to this, the specific activity of ALP was higher in the bound fraction, but substantial variability was observed between different isolates. It should be borne in mind that this is comparison of two different Con A-resolved fractions (their yield and purity can vary independently). In general, ALP activity can be expected to be more sensitive to experimental conditions or influences by aggregation (common to concentration and ultracentrifugation steps) than GGT. Regarding WGA-separated fractions, GGT had almost the same specific activity in non-bound and bound fractions, whereas almost all ALP remained non-bound. Thus, ALP activity was very low/undetectable in WGA-bound fractions. In addition, it was always higher in the Con A-bound than in the Con A-non-bound fraction. Thus, both of these references were reproducible for Con A-bound fractions of sPro-N samples, resulting in its annotation as ALP-associated. The exosome marker, CD63, was clearly detected in the non-bound fractions separated on both Con A and WGA columns as well as in WGA-bound fractions, i.e. it overlapped the GGT distribution by not varying between different isolates. However, CD63 immunoreactivity was much less intense or hardly visible in the Con A-bound fraction (divergence between different isolates).

**Table 1.** Surface-associated markers of seminal prostasomes from normozoospermic men separated by lectin-affinity chromatography.

Column	LAC-separated fractions	sPro-N1^a^			sPro-N2^a^		
		GGT (U/g protein)^b^	ALP (U/g protein)^b^	CD63^c^	GGT (U/g protein)^b^	ALP (U/g protein)^b^	CD63^c^
Con A	Non-bound	513	46	+++	612.3	10.1	+++
	Bound	102.6	54.8	+	127.3	316.7	+/−
WGA	Non-bound	294.4	31.6	+++	371.7	6.8	+++
	Bound	242.4	2.8	++	208.2	0	++

a sPro-N1 and sPro-N2: different isolates of seminal prostasomes from normozoospermic men.

b Enzyme activity (U/L) was normalised to concentration of proteins (g/L) in corresponding fraction.

c CD63: immunoreactivity as determined by dot-blot (+++ intense; ± low/hardly visible).

ALP: alkaline phosphatase; GGT: gamma-glutamyl transferase.

**Figure 3.**
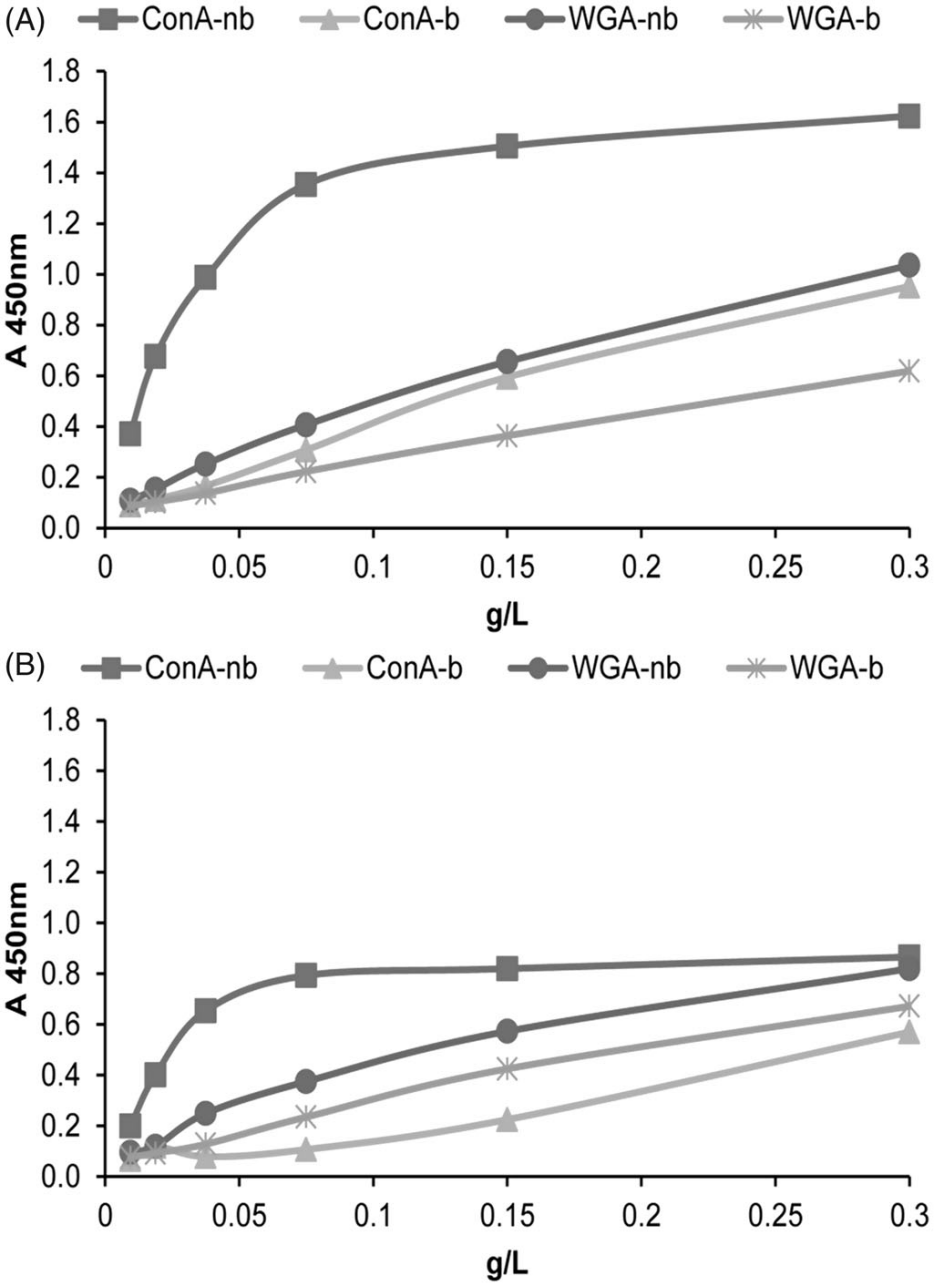
Surface glycosylation of seminal prostasomes from normozoospermic men separated by lectin-affinity chromatography (LAC). LAC-separated fractions of seminal prostasomes from normozoospermic men: Con A-non-bound (Con A-nb), Con A-bound (Con A-b), WGA-non-bound (WGA-nb), and WGA-bound (WGA-b) fractions were immobilized and re-probed with Con A (A) and WGA (B) by solid-phase binding assay.

In summary, the results obtained indicated two/three CD63-positive populations which differed slightly in GGT activity and had the typical prostasome electrophoretic pattern. Since they were separated into Con A-/WGA-non-bound populations and the WGA-bound one, it is possible that they differ in the presentation/density of shared surface glycans. This was tested using Con A and WGA lectins as tracers in the fluid phase (Figure 3).

Indeed, when probed as an unfractionated sample, sPro-N exhibited strong binding to both Con A and WGA (data not shown). However, this changed after separation by LAC on the Con A and WGA columns in a way that supported enrichment of distinct populations from the pre-existing mixture of vesicles, some of which remained different in the related lectin non-bound populations.

Thus, Con A-binding curves indicated that all LAC-separated fractions are reactive (Figure 3(A)). Referring to the CD63-immunoreactive populations, each of two resolved on the WGA column exhibited lower reactivity compared to the one (containing almost all CD63 immunoreactivity) resolved as non-bound on the Con A column. However, when referring to lectin-binding as such, both the Con A-non-bound fraction and the Con A-bound fraction exhibited opposite reactivity when they were immobilized (solid-phase assay) from that when they were in the fluid phase (LAC). This may be due to known influence of ligand orientation on affinity of lectins. However, it should be taken into account that Con A-resolved fractions (by LAC), which were re-checked in the solid-phase assay, were found to differ judging by their electrophoretic pattern, CD63, ALP, and GGT. Thus, surface assembly and accessibility of Con A-binding moieties (on competing Con A-reactive entities) seem to be very demanding in terms of their separation than WGA-binding moieties. This notion may be supported by the matching patterns of WGA-binding curves (Figure 3(B)) indicating that all LAC-separated fractions are reactive and similar, regardless of the column used.

### Lectin-affinity chromatography of seminal prostasomes from oligozoospermic men

**Figure 4.**
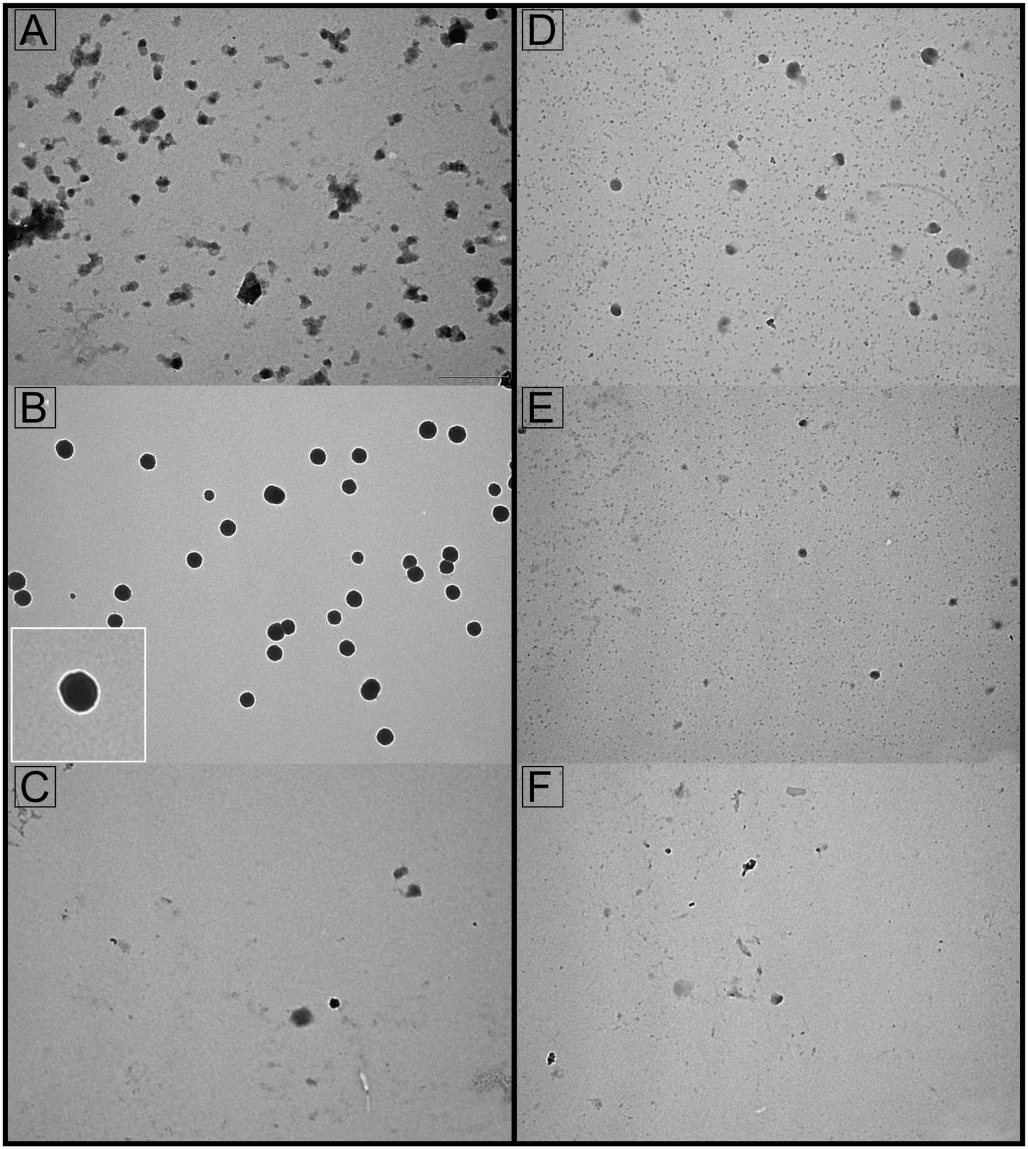
Transmission electron microscopy of seminal prostasomes from oligozoospermic men separated by lectin-affinity chromatography. Micrographs of vesicles (70–120 nm) in a concanavalin A (Con A)-non-bound fraction (A) and vesicles (80–170 nm) in a Con A-bound fraction (B). The insert shows the typical cup-shape appearance enlarged. Micrographs of vesicles (70–130 nm) in a wheat germ agglutinin (WGA)-non-bound fraction (D) and vesicles (50–100 nm) in a WGA-bound fraction (E). Vesicles in the high-pH-eluted fraction from Con A column (C) and low-pH-eluted fraction from WGA column (F) were scarce.

Seminal prostasomes from oligozoospermic men (sPro-O) resolved by Con A and WGA affinity chromatography distributed across non-bound and bound fractions exhibited no striking difference at the ultrastructural level (Figure 4).

**Figure 5.**
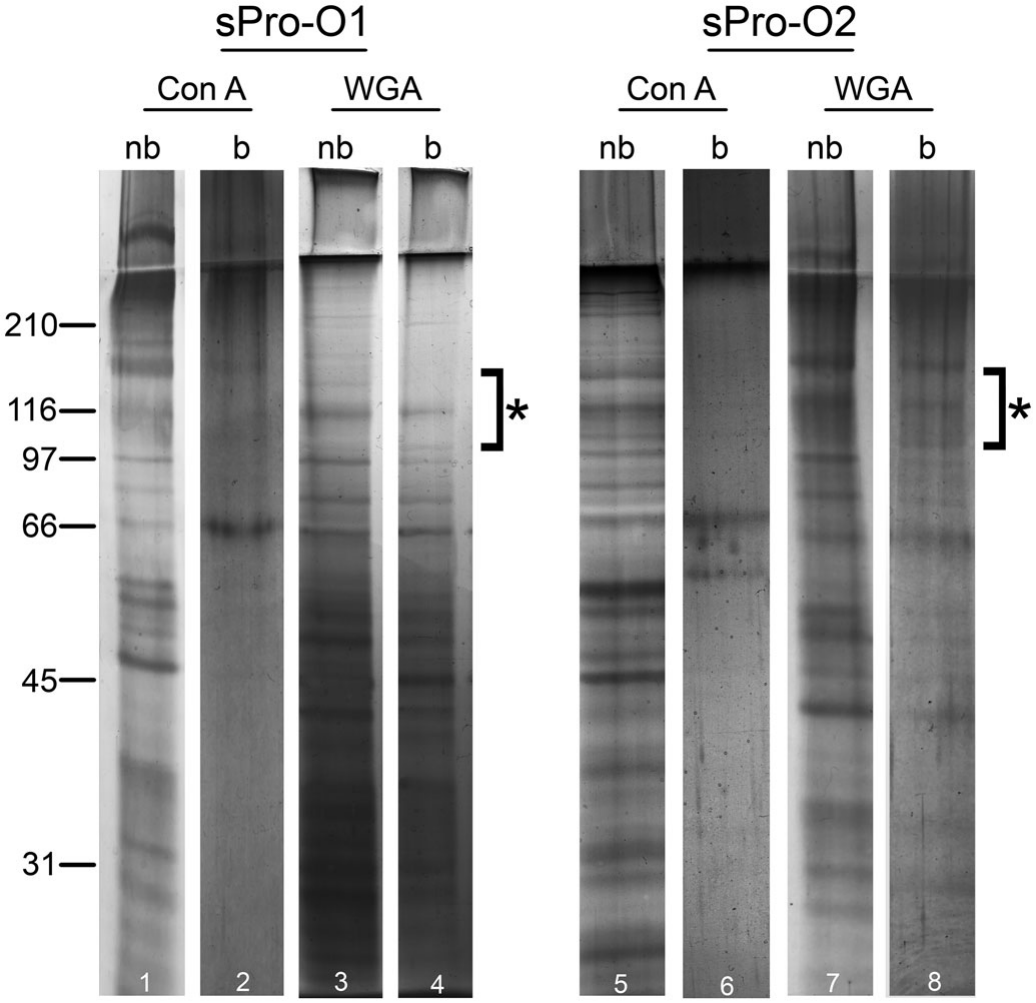
Protein composition of seminal prostasomes from oligozoospermic men separated by lectin-affinity chromatography. Seminal prostasomes separated by lectin-affinity chromatography were resolved on 10% SDS-PAGE under reducing and denaturing conditions and stained with silver. Representative total protein patterns of non-bound (nb) and bound (b) fractions separated on a Con A column and on a WGA column were shown. Samples were loaded as isolated (equal volume/line to reflect yield), and total protein amounts were: line 1 (19 μg), line 2 (10 μg), line 3 (14 μg), line 4 (11 μg), line 5 (50 μg), line 6 (6 μg), line 7 (24 μg), line 8 (13 μg). Characteristic prostasome-associated bands in the region of 90–150 kDa are marked (asterisk). Numbers indicate the position of molecular mass standards (kDa). sPro-O1 and sPro-O2: different isolates of seminal prostasomes from oligozoospermic men.

Non-bound fractions separated on Con A (Figure 5, lines 1–5) and WGA columns (Figure 5, lines 3–7) as well as the WGA-bound fraction (Figure 5, lines 4–8) all revealed similar electrophoretic patterns including diverse prostasome-specific bands at 150–90 kDa. However, differences could be observed regarding bands below 66 kDa. In the Con A-bound fraction, there was a prostasome signature band, at 150 kDa, but any others were undetectable (Figure 5, lines 2–6).

The specific activity of GGT in Con A-separated fractions was much higher in the non-bound than in the sugar-eluted fraction (Table 2). In contrast to this, ALP-specific activity was higher in the bound fraction compared to the non-bound fraction (divergence between different isolates). Concerning WGA-separated fractions, all ALP-specific activity remained in the non-bound fraction, whereas GGT-specific activity was higher than in sugar-eluted fraction. CD63 was detected in the non-bound fractions separated on both Con A and WGA columns, as well as in the WGA-bound fraction, but it was low or hardly detectable in the Con A-bound fraction.

**Table 2.** Surface-associated markers of seminal prostasomes from oligozoospermic men separated by lectin-affinity chromatography.

Column	LAC-separated fractions	sPro-O1^a^			sPro-O2^a^		
		GGT (U/g protein)^b^	ALP (U/g protein)^b^	CD63^c^	GGT (U/g protein)^b^	ALP (U/g protein)^b^	CD63^c^
Con A	Non-bound	389.5	50.8	+++	89.2	11.3	+++
	Bound	35.1	181.2	+	19.3	256	–
WGA	Non-bound	235.5	20.8	+++	345	10.7	+++
	Bound	102.2	0	++	154.1	0	++

a sPro-O1 and sPro-O2: different isolates of seminal prostasomes from oligozoospermic men.

b Enzyme activity (U/L) was normalised to concentration of proteins (g/L) in corresponding fraction.

c CD63: immunoreactivity as determined by dot-blot (+++ intense; ± low/hardly visible).

ALP: alkaline phosphatase; GGT: gamma-glutamyl transferase.

**Figure 6.**
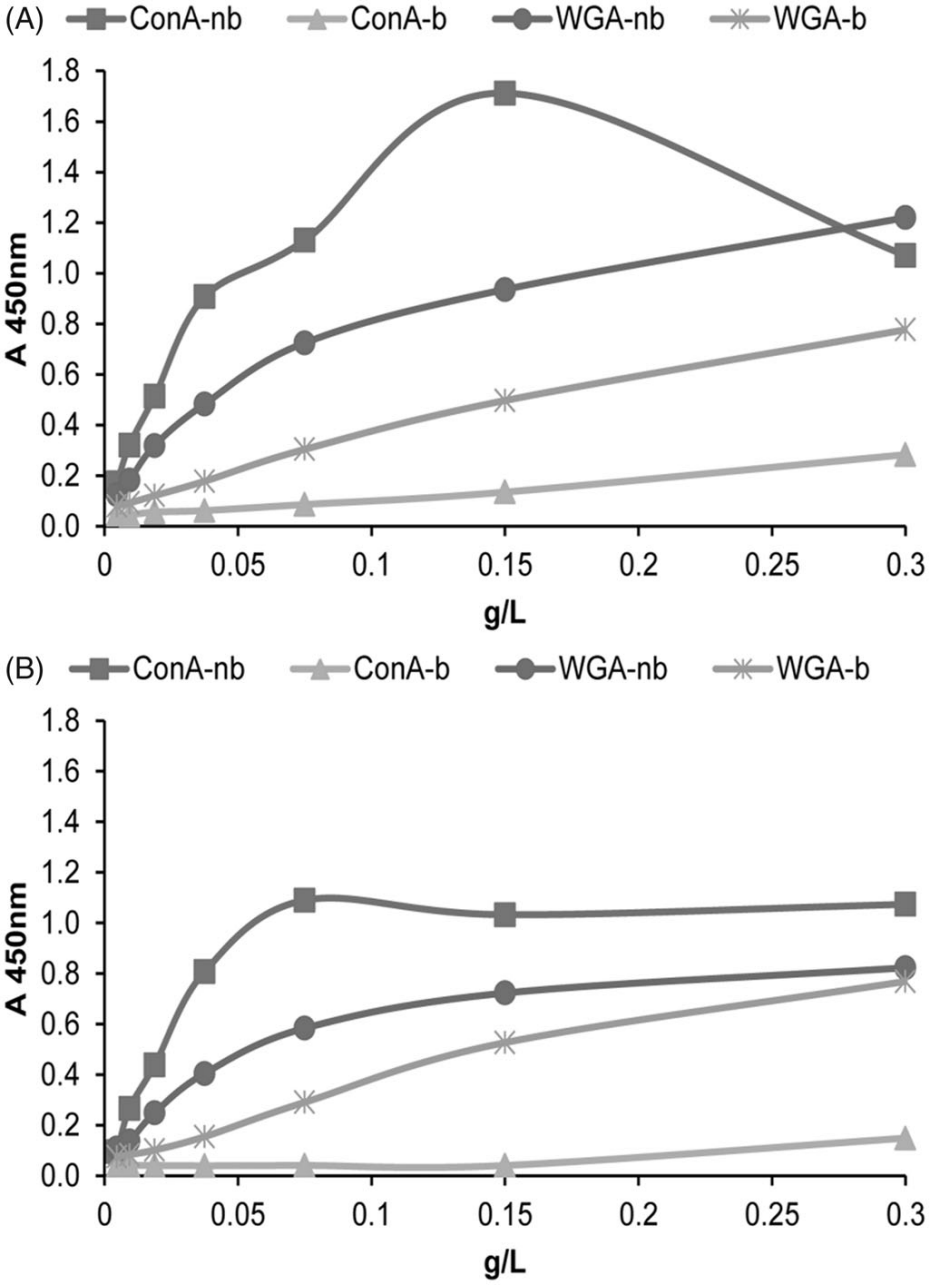
Surface glycosylation of seminal prostasomes from oligozoospermic men separated by lectin-affinity chromatography. LAC-separated fractions of seminal prostasomes from oligozoospermic men: Con A-non-bound (Con A-nb), Con A-bound (Con A-b), WGA-non-bound (WGA-nb), and WGA-bound (WGA-b) fractions were immobilized and re-probed with Con A (A) and WGA (B) by solid-phase binding assay.

Con A (Figure 6(A)) and WGA reactivity (Figure 6(B)) of LAC-separated fractions indicated that CD63-immunoreactive populations exhibited comparable lectin-binding curves to both lectins.

However, when referring to lectin-binding itself, both the Con A-non-bound fraction and the Con A-bound fraction exhibited opposite reactivity to that expected, as was also observed for sPro-N (Figure 3). In addition, this effect was more pronounced in sPro-O and greatly affected the related Con A-bound population.

In summary, the results obtained indicated that sPro-O was separated on Con A and WGA columns, in the same way as sPro-N. However, the corresponding populations of sPro-N (Figure 2) and sPro-O, which have the typical prostasome electrophoretic pattern, differed regarding GGT activity. Moreover, related sPro-N and sPro-O populations tracked by ALP activity were strikingly different in respect to presentation of surface glycans.

## Discussion

Concerning the importance of choosing appropriate protocols for obtaining specified preparations of EVs, this study has pointed to the usability/suitability of LAC as an adjunct tool to complement standard procedures for separating and closely annotating vesicle populations. Work with glycans means coping with their intrinsic heterogeneity and requirements for binding to the matrix posed by their spatial orientation, density, and the accessibility of distinct moieties, without regard to the examined source (28,29). In relation to this, checking surface glycans is the initial step preceding affinity chromatography that is necessary for determining which lectins to use. Consequently, the lectins applied here for separation of seminal prostasomes are not in any way demonstrated to be appropriate for other vesicles, but the principles of utilizing LAC are valid. In this study, Con A and WGA were chosen as the reference capturing agents (20,21,30). In general, for affinity chromatography as self-contained or as serial, Con A and WGA, which differ in their carbohydrate-binding specificities, are usually combined to gain insight into the presence of distinct N-linked oligosaccharide chains (31,32). Moreover, we used them in a solid-phase binding assay to re-check non-bound and bound vesicle populations, since it is known that the affinity of lectins for the same glycans (differently accessible on distinct populations) can vary depending on whether the phase is fluid or stationary (33). Thus, vesicles are complex systems representing particles on which structurally related glycans can have different presentations due to the density and nature of their carriers. After separation, their detection, i.e. lectin-binding, may be changed due to alterations in the exposure of sugar moieties.

Starting from this, we established Con A-/WGA-binding as a new reference parameter and also rediscovered the prostasomal surface-associated glycoprotein enzymes GGT/ALP, all by monitoring separation of vesicle subpopulations. Having in mind that chromatographic techniques in exosome research have shortcomings in terms of low yield and that separation is not clear cut (34,35), we evaluated the general patterns of distribution/relative ratios (not absolute values) of these selected markers in bound and non-bound fractions.

The results obtained indicated that both sPro-N and sPro-O consist of two CD63-immunoreactive populations exhibiting typical prostasome protein signature bands, but they differed in specific activity of associated enzymes and the presentation of surface glycans. WGA seems to be the lectin of choice for their separation since, when passed through the Con A column, the prostasomal patterns of glycoproteins and selected markers remained mainly in the non-bound fraction. Nevertheless, there were Con A-bound populations, the pattern of which lacks some common prostasome bands. They can be tracked down by the specific activity of ALP, in both sPro-N and sPro-O. Moreover, the solid-phase lectin-binding assay indicated striking differences in the presentation of their Con A-binding moieties. It is possible that the Con A-reactive vesicles represent other non-prostasomes/lipid vesicles or one more distinct prostasome population, but at this stage we cannot resolve this issue (20).

In general, the distribution and/or co-distribution of individual markers may reflect differences in their accessibility on vesicle surfaces. Thus, data on the nature of association of GGT and ALP with vesicles may coincide with specific association of markers on distinct vesicle populations. It has been shown that GGT co-localizes in lipid rafts with tetraspanins (36) and can be released from different lipoparticle complexes, including prostasomes, by proteolytic cleavage with papain without or after pre-treatment with detergents (22). In contrast to this, ALP is supposed to be linked to the prostasome membrane via a glycosylphosphatidylinositol (GPI) anchor, as it can be released only by phosphoinositol-specific phospholipase C (24).

EVs are intrinsically complex and heterogeneous. Constant improvements of current protocols for their characterization are needed together with the introduction of new approaches (8–10,37). Altogether, our findings regarding seminal prostasomes using LAC confirmed the heterogeneity found with other methods (1,3,4,38). In addition, they, also, indicated differences between seminal prostasomes from normozoospermic and oligozoospermic men as observed in other studies (20,39,40). They justify further technical work regarding experimental design in terms of scalability/improved yield for general preparative vesicle purification/isolation using lectin-affinity capture. This was outside the scope of this introductory step with LAC. From basic aspects, the results obtained provided new qualitative data in terms of annotation of selected glyco-parameters for distinct prostasome population(s). Specifically they draw attention to the possible significance of surface composition including association with enzyme activity in differentiating between vesicles without examining their cargo or looking for particular cell-specific proteins as possible recognition badges. In addition, a new avenue emerged for the use of prostasome-associated GGT and ALP, known indicators of prostate secretory activity (41–43) as biomarkers.

## Abbreviations

EVs: extracellular vesicles
SP: seminal plasma
LAC: lectin-affinity chromatography
sPro-N: seminal prostasomes from normozoospermic men
sPro-O: seminal prostasomes from oligozoospemic men
Con A: concanavalin A
WGA: wheat germ agglutinin
GGT: gamma-glutamyl transferase
ALP: alkaline phosphatase
TMB- 3,3′,5,5′: tetramethylbenzidine
HRP: horseradish peroxidase
GPI: glycosylphosphatydilinosito

## References

[1] Carlsson L, Nilsson O, Larsson A, Stridsberg M, Sahlén G, Ronquist G Characteristics of human prostasomes isolated from three different sources. Prostate. 2003;54:322–30. doi:10.1002/pros.10189 12539232

[2] Sahlén G, Nilsson O, Larsson A, Carlsson L, Norlén BJ, Ronquist G Secretions from seminal vesicles lack characteristic markers for prostasomes. Ups J Med Sci. 2010;115:107–12. doi:10.3109/03009730903366067 19943818 PMC2853787

[3] Ronquist G Prostasomes are mediators of intercellular communication: from basic research to clinical implications. J Intern Med. 2012;271:400–13. doi:10.1111/j.1365-2796.2011.02487.x 22112042

[4] Aalberts M, Stout TAE, Stoorvogel W Prostasomes: extracellular vesicles from the prostate. Reproduction. 2014;147:R1–14. doi:10.1530/REP-13-0358 24149515

[5] György B, Szabó TG, Pásztói M, Pál Z, Misják P, Aradi B, et al. Membrane vesicles, current state-of-the-art: emerging role of extracellular vesicles. Cell Mol Life Sci. 2011;68:2667–88. doi:10.1007/s00018-011-0689-3 21560073 PMC3142546

[6] Kim D, Nishida H, An SY, Shetty AK, Bartosh TJ, Prockop DJ Chromatographically isolated CD63 + CD81+ extracellular vesicles from mesenchymal stromal cells rescue cognitive impairments after TBI. Proc Natl Acad Sci USA. 2016;113:170–5. doi:10.1073/pnas.1522297113 26699510 PMC4711859

[7] Kowal J, Arras G, Colombo M, Jouve M, Morath JP, Primdal-Bengtson B, et al. Proteomic comparison defines novel markers to characterize heterogeneous populations of extracellular vesicle subtypes. Proc Natl Acad Sci USA. 2016;113:E968–77. doi:10.1073/pnas.1521230113 26858453 PMC4776515

[8] Gardiner C, Di Vizio D, Sahoo S, Théry C, Witwer KW, Wauben M, et al. Techniques used for the isolation and characterization of extracellular vesicles: results of a worldwide survey. J Extracell Vesicles. 2016;5:32945. doi:10.3402/jev.v5.32945 27802845 PMC5090131

[9] Yáñez-Mó M, Siljander PRM, Andreu Z, Zavec AB, Borràs FE, Buzas EI, et al. Biological properties of extracellular vesicles and their physiological functions. J Extracell Vesicles. 2015;4:27066. doi:10.3402/jev.v4.27066 25979354 PMC4433489

[10] Fais S, O’Driscoll L, Borras FE, Buzas E, Camussi G, Cappello F, et al. Evidence-based clinical use of nanoscale extracellular vesicles in nanomedicine. ACS Nano. 2016;10:3886–99. doi:10.1021/acsnano.5b08015 26978483

[11] Kalra H, Drummen GPC, Mathivanan S Focus on extracellular vesicles: introducing the next small big thing. IJMS. 2016;17:170. doi:10.3390/ijms17020170 26861301 PMC4783904

[12] Batista BS, Eng WS, Pilobello KT, Hendricks-Muñoz KD, Mahal LK Identification of a conserved glycan signature for microvesicles. J Proteome Res. 2011;10:4624–33. doi:10.1021/pr200434y 21859146 PMC3443565

[13] Staubach S, Schadewaldt P, Wendel U, Nohroudi K, Hanisch FG Differential glycomics of epithelial membrane glycoproteins from urinary exovesicles reveals shifts toward complex-type N-glycosylation in classical galactosemia. J Proteome Res. 2012;11:906–16. doi:10.1021/pr200711w 22087537

[14] Gerlach JQ, Krüger A, Gallogly S, Hanley SA, Hogan MC, Ward CJ, et al. Surface glycosylation profiles of urine extracellular vesicles. PLoS One. 2013;8:e74801. doi:10.1371/journal.pone.0074801 24069349 PMC3777961

[15] Raposo G, Stoorvogel W Extracellular vesicles: exosomes, microvesicles, and friends. J Cell Biol. 2013;200:373–83. doi:10.1083/jcb.201211138 23420871 PMC3575529

[16] Colombo M, Moita C, van Niel G, Kowal J, Vigneron J, Benaroch P, et al. Analysis of ESCRT functions in exosome biogenesis, composition and secretion highlights the heterogeneity of extracellular vesicles. J Cell Sci. 2013;126:5553–65. doi:10.1242/jcs.128868 24105262

[17] Liang Y, Eng WS, Colquhoun DR, Dinglasan RR, Graham DR, Mahal LK Complex N-linked glycans serve as a determinant for exosome/microvesicle cargo recruitment. J Biol Chem. 2014;289:32526–37. doi:10.1074/jbc.M114.606269 25261472 PMC4239607

[18] Buzás EI, Tóth E, Sódar BW, Szabó-Taylor K Molecular interactions at the surface of extracellular vesicles. Semin Immunopathol. 2018;40:453–64. doi:10.1007/s00281-018-0682-0 29663027 PMC6208672

[19] Lässer C, Jang SC, Lötvall J Subpopulations of extracellular vesicles and their therapeutic potential. Mol Aspects Med. 2018;60:1–14. doi:10.1016/j.mam.2018.02.002 29432782

[20] Milutinović B, Goč S, Mitić N, Kosanović M, Janković M Surface glycans contribute to differences between seminal prostasomes from normozoospermic and oligozoospermic men. Ups J Med Sci. 2019;124:111–8. doi:10.1080/03009734.2019.1592266 30957617 PMC6566730

[21] Milutinovic B, Mitic N, Roncevic J, Goc S, Jankovic M Glycome complexity of human seminal plasma high molecular mass components: evaluation of the contribution of acid-soluble glycoproteins/mucins and extracellular vesicles. Arch Biochem Biophys. 2016;609:20–30. doi:10.1016/j.abb.2016.09.005 27639309

[22] Lilja H, Weiber H γ-Glutamyltransferase bound to prostatic subcellular organelles and in free form in human seminal plasma. Scand J Clin Lab Invest. 1983;43:307–12. doi:10.3109/00365518309168262 6138849

[23] Fabiani R Functional and biochemical characteristics of human prostasomes. Ups J Med Sci. 1994;99:73–111. doi:10.3109/03009739409179353 7716832

[24] Fabiani R, Ronquist G Association of some hydrolytic enzymes with the prostasome membrane and their differential responses to detergent and PIPLC treatment. Prostate. 1995;27b:95–101. doi:10.1002/pros.2990270206 7638087

[25] Mitić N, Kosanović M, Milutinović B, Goč S, Mladenović D, Grubiša I, et al. Nano-sized CA125 antigen glycocamouflage: mucin - extracellular vesicles alliance to watch? Arch Biochem Biophys. 2018;653:113–20. doi:10.1016/j.abb.2018.06.017 29969582

[26] Kosanović M, Milutinović B, Goč S, Mitić N, Janković M Ion-exchange chromatography purification of extracellular vesicles. Biotechniques. 2017;63:65–71. doi:10.2144/000114575 28803541

[27] Laemmli UK Cleavage of structural proteins during the assembly of the head of bacteriophage T4. Nature. 1970;227:680–5. doi:10.1038/227680a0 5432063

[28] Moremen KW, Tiemeyer M, Nairn AV Vertebrate protein glycosylation: diversity, synthesis and function. Nat Rev Mol Cell Biol. 2012;13:448–62. doi:10.1038/nrm3383 22722607 PMC3934011

[29] Brockhausen I, Glycobiology protocols. Totowa, NJ: Humana Press; 2006.

[30] Ronquist G, Hedström M Restoration of detergent-inactivated adenosine triphosphatase activity of human prostatic fluid with concanavalin A. Biochim Biophys Acta. 1977;483:483–6. doi:10.1016/0005-2744(77)90078-X 142513

[31] Sumi S, Arai K, Kitahara S, Yoshida K Serial lectin affinity chromatography demonstrates altered asparagine-linked sugar-chain structures of prostate-specific antigen in human prostate carcinoma. J Chromatogr B Biomed Sci Appl. 1999;727:9–14. doi:10.1016/S0378-4347(99)00069-9 10360417

[32] Cummings RD Lectins as tools for glycoconjugate purification and characterization In: Gabius H-J, Gabius S Glycosciences: status and perspectives. London, UK: Chapman and Hall; 2008 191–9.

[33] Tang W, Miura T, Nakata M, Kojima N, Mizuochi T Binding specificities of lectins to immobilized glycoproteins and oligosaccharides differ from those of immobilized lectins to oligosaccharides. Acta Med Okayama. 1998;52:311–8. doi:10.18926/AMO/31309 9876768

[34] Balaj L, Atai NA, Chen W, Mu D, Tannous BA, Breakefield XO, et al. Heparin affinity purification of extracellular vesicles. Sci Rep. 2015;5:10266. doi:10.1038/srep10266 25988257 PMC4437317

[35] Wang T, Turko IV Proteomic toolbox to standardize the separation of extracellular vesicles and lipoprotein particles. J Proteome Res 2018;17:3107–13. 10.1021/acs.jproteome.8b00225 PMC7307575 30080417

[36] Dubois L, Ronquist KG, Ek B, Ronquist G, Larsson A Proteomic profiling of detergent resistant membranes (lipid rafts) of prostasomes. Mol Cell Proteomics. 2015;14:3015–22. doi:10.1074/mcp.M114.047530 26272980 PMC4638043

[37] Witwer KW, Buzás EI, Bemis LT, Bora A, Lässer C, Lötvall J, et al. Standardization of sample collection, isolation and analysis methods in extracellular vesicle research. J Extracell Vesicles. 2013;2:20360. doi:10.3402/jev.v2i0.20360 PMC3760646 24009894

[38] Poliakov A, Spilman M, Dokland T, Amling CL, Mobley JA Structural heterogeneity and protein composition of exosome-like vesicles (prostasomes) in human semen. Prostate. 2009;69:159–67. doi:10.1002/pros.20860 18819103

[39] Brody I, Ronquist G, Gottfries A, Stegmayr B Abnormal deficiency of both Mg2+ and Ca2+-dependent adenosine triphosphatase and secretory granules and vesicles in human seminal plasma. Scand J Urol Nephrol. 1981;15:85–90. doi:10.3109/00365598109179581 6120566

[40] García-Rodríguez A, de la Casa M, Peinado H, Gosálvez J, Roy R Human prostasomes from normozoospermic and non-normozoospermic men show a differential protein expression pattern. Andrology 2018;6:585–96. doi:10.1111/andr.12496 29726126

[41] Verhoeven G, Steeno O Evidence for the prostatic origin of gamma-glutamyltransferase activity in human semen. Andrologia. 1979;1:163–9. doi:10.1111/j.1439-0272.1979.tb02181.x 36011

[42] Frierson HJ, Theodorescu D, Mills S, Hanigan M Gamma-glutamyl transpeptidase in normal and neoplastic prostate glands. Mod Pathol 1997;10:1–6. 9021720

[43] Whitfield JB Gamma glutamyl transferase. Crit Rev Clin Lab Sci. 2001;38:263–355. doi:10.1080/20014091084227 11563810

